# Anithrombotic prevention in vascular disease: bases for a new strategy in antithrombotic therapy

**DOI:** 10.1186/1477-9560-5-11

**Published:** 2007-08-29

**Authors:** Raul Altman

**Affiliations:** 1 Centro de Trombosis de Buenos Aires, Buenos Aires, Argentina; 2 Cátedra of Master in Thrombosis and Unidad Docente Buenos Aires, Faculty of Medicine, National University of Tucumán, Argentina

## Abstract

A tendency toward bleeding often undercuts the beneficial preventive effect of higher doses of a single antithrombotic drug or combined antithrombotic therapy. Although high doses of antithrombotic drugs may be necessary for optimal prevention, such therapy can also elicit more frequent bleeding. Although major bleeding could be a reversible event is likely to lead clinicians to discontinue antithrombotic therapy which in turn could increase the risk of myocardial infarction, stroke, and cardiovascular death. Thus, to prevent thrombotic events without frequent bleeding complications, the preferred approach might be to use anti-inflammatory drugs in addition to the first-line antithrombotic drugs to reduce inflammation and thrombin formation in atheroma. Although some preliminary data have been already published, to confirm the potential benefit of anti-inflammatory drugs in acute coronary syndromes large prospective double-bind randomized trials are necessary.

## Background

The hemostatic system is a physiological process to prevent hemorrhage and maintain a balance between clot formation and fluidity of blood in the circulation. After endothelial injury, platelets adhere to the exposed subendothelium and are activated by locally released agonists to stimulate thrombin formation [1], contributing to hemostatic control. Under other circumstances, such as in arterial disease, clots form upon atheroma rupture, primarily in connection with plaque conditions and in situ thrombin generation. After a clot starts to form, its growth depends on platelet recruitment and aggregation, concentration of thrombin at the surface of the clot, and changes in local blood flow. Thus, clot growth can be inhibited by blocking thrombus-bound thrombin activity and/or by inhibiting platelet function.

Thrombin is the strongest platelet agonist, and inhibition of thrombin can also prevent platelet activation. In fact, any intervention singly directed at thrombin activity or platelet activation may simultaneously affect the other function [2].

Aspirin (acetylsalicylic acid), and clopidogrel, are the mainstay antiplatelet therapies for arterial disease. Together with heparin, they constitute the first-line treatments for acute coronary disease. Clinical trials have established the benefit of aspirin for coronary prevention, and it is considered the gold standard for arterial antithrombotic therapy – despite its limitations with regard to preventing thrombosis.

Coadministration of aspirin and clopidogrel enhances platelet inhibition, because these agents act through different platelet receptors; however, the benefit of combined antiplatelet treatment over aspirin alone in preventing thrombotic outcomes is debated [3-5].

Therapies that include multiple drugs affecting primary hemostasis (antiplatelet drugs) or a combination of antiplatelets and anticoagulants, as in ischemic heart disease, are more aggressive than a single therapy. Combined antithrombotic treatment confers particular risk and is associated with a higher incidence of bleeding [6,7], especially among older patients and patients undergoing early revascularization [8]. The combining therapy, of clopidogrel and aspirin, causes more bleeding than monotherapy with either drug [9].

In arterial thrombosis, which mainly occurs on atheroma, inflammation promotes atheroma rupture. Thus there is a need for an alternative to high doses of antithrombotic drugs and that evidence regarding the effectiveness of anti-inflammatory agents will be discussed.

### Thrombosis versus hemostasis mechanisms

Current speculation is that the pathways for hemostasis may differ from the pathways of thrombosis, as it is possible to attenuate thrombosis without inhibiting hemostasis [10,11].

Theoretically, the risk of thrombotic events may be increased by the exposure of procoagulant tissue factor (TF) to the circulation. The presence of TF on the outer membrane is a prominent characteristic of plaque lesions. Macrophages/foam cells, smooth muscle cells, and apoptotic cells are sources of exposed TF. In patients with a disrupted atheroma that contains abundant TF, there is increased generation of surrounding thrombin. Procoagulant microparticles from stimulated and apoptotic cells may also contribute to acute thrombotic occlusion [12]. Microparticle tissue factor appears to play a dominant role in fibrin generation and thrombus propagation but tissue factor in blood may be encrypted so as not to cause thrombosis in the absence of specific stimuli [13].

Thrombus formation is driven primarily by TF derived from the vessel wall [14] and circulating TF contributes to thrombosis, but is unlikely to play a significant role in hemostasis [15].

Hemostasis normally occurs in minutes, and a small amount of blood-borne TF can accumulate at the site of injury. In contrast, thrombosis may occur over the course of minutes or hours, and it involves large volumes of blood flow, which permits accumulation of significant amounts of blood-borne TF.

Decryption of platelet TF is linked to membrane phosphatidylserine. After undergoing the flip-flop process, platelet membrane phosphatidylserine can cause decryption of platelet TF and may contribute to local hemostasis, although the potential importance of this action is not well defined [16]. Platelets activated by factor VIIa and other agonists (arachidonic acid, collagen, and ADP) can induce thrombin generation independent of external TF and may be important in normal hemostasis [1].

Hemostasis is a local not progressive process because clotting starts when 10–20 nmol/l of thrombin is formed; this initial thrombin activity is important for hemostasis [17]. The local clotting mechanism is a process limited by several factors such as the small amount of exposed TF in a relatively small area of the vessel wall, dilution of activators in the bloodstream of non occluded vessels and it inhibition by tissue factor pathway inhibitor (TFPI) and other natural coagulation inhibitors, and restraint of clot lysis by platelet plasminogen activator inhibitor, which prevents the release of clot-bound thrombin.

In arterial thrombosis, which mainly occurs on atheroma, inflammation promotes atheroma rupture. In atheroma, the levels of TF, which is also expressed by monocytes and macrophage-derived foam cells, are several-fold greater than the levels in a wounded vessel [18]. Enhancement of the TF/FVIIa complex leads to thrombin generation and strong activation of platelets. Blood flow changes (stasis) in a partially or fully occluded vessel prevent the dilution of surrounding activated factors and clot-bound thrombin and, together with platelet-erythrocyte interaction and thrombin-activatable fibrinolysis inhibitor (TAFI), promote thrombus growth. Elevated levels of TAFI in coronary heart disease are associated with risk of arterial thrombosis [19]. Also, in unstable angina and silent ischemia, local thrombolysis releases fibrin-bound thrombin, favoring thrombus growth [20].

### Dose-ranging studies of antithrombotic agents

Numerous randomized controlled trials have evaluated the effectiveness and safety of anticoagulants or antiplatelet therapy or different combinations of anticoagulant and antiplatelet therapy; or dual antiplatelet therapy were compared with aspirin in short or long-term thrombosis prevention studies.

In a dose-ranging trial of enoxaparin 1.25 mg/kg and 1.0 mg/kg (every 12 hr) in patients with unstable angina, the incidence of death, recurrent myocardial infarction, or recurrent ischemia requiring revascularization was 5.6% with the higher dose and 5.2% with the lower dose [21]. Major hemorrhage occurred in 1.9% of patients in the 1.0 mg/kg group and 6.5% in the 1.25 mg/kg group (p = 0.03). Thus, lower dose achieved equivalent risk reduction with less bleeding [21]. Similarly, in the Instability in Coronary Artery Disease (FRISC) study where another low molecular weight heparin fragmin was tested, the incidence of major bleeding was 6% with a dose of 150 IU/kg every 12 hr and 0.8% with a dose of 120 IU/kg every 12 hr [22].

Melagatran and dabigatran are active antithrombin drugs that selectively and competitively inhibit free and clot-bound thrombin. After oral administration, ximelagatran is absorbed and biotransformed to melagatran, the dominant compound in plasma. In a dose-ranging study comparing melagatran/ximelagatran to the low molecular weight dalteparin, the highest dose of ximelagatran had better clinical efficacy and a nonsignificant tendency toward a greater transfusion volume in hip replacement patients [23]. Although there were no differences in recurrent venous thromboembolism events during 6 months of treatment and besides hepatotoxicity events, serious symptomatic myocardial ischemia warranting hospitalization was referred by the use of ximelagatran.

Another study reported that total bleeding (major and minor) was lower with ximelagatran versus warfarin in atrial fibrillation patients [24]. However, other work also in nonvalvular atrial fibrillation patients demonstrated a small dose-related increase in bleeding with ximelagatran [25].

In dose-ranging studies with dabigatran, another active antithrombin drug, in patients undergoing hip or knee surgery [26], there was a significant dose-dependent decrease in the frequency of venous thromboembolism (VTE) with increasing doses of dabigatran etexilate (the prodrug of dabigatran). The lowest rate of total VTE (13.1%) and proximal deep venous thrombosis DVT (1.7%) occurred in the highest dose group (225 mg twice daily). There was also a dose-related increase in major bleeding episodes. Hemorrhagic events were significantly more frequent with higher doses versus the lowest dose of dabigatran (3.8% and 0.3%, respectively). The combined clinically significant bleeding and major hemorrhage followed the same pattern: 8.4% with the 225 mg dose and 2.6% with the 50 mg dose. The increase in bleeding was irrespective of the surgical procedure.

A dose-ranging study of BAY 59-7939 (Rivaroxaban), a direct oral inhibitor of activated Factor X, was performed with total knee replacement patients [27]. Among the five BAY 59-7939 dose groups tested (2.5 mg, 5 mg, 10 mg, 20 mg, and 30 mg twice daily), the incidence of the primary efficacy endpoint ranged from 23.3% (10 mg twice daily) to 40.4% (5 mg twice daily). There was a trend toward efficacy with the higher dose, although it was not significant based on the total daily dose (p = 0.29). However, there was a statistically significant dose-related increase in postoperative major bleeding episodes (1.0%, 0%, 1.9%, 3.1%, and 7.5% for 2.5 mg, 5 mg, 10 mg, 20 mg, and 30 mg twice daily, respectively; p = 0.0007). Mean transfusion volumes were lowest for patients receiving 2.5 mg and highest for patients receiving 30 mg. In a second dose-finding study of BAY 59-7939 in total hip replacement patients [28], there was a significant dose-dependent trend, with the highest dose yielding better efficacy than the lowest dose. Major postoperative bleeding was significant and dose-related (0.8%, 2.2%, 2.3%, 4.5%, and 5.4% for 2.5 mg, 5 mg, 10 mg, 20 mg, and 30 mg BAY 59-7939 twice daily, respectively).

A third dose-ranging study with BAY 59-7939 administered as a single daily dose was recently published [29]. Results in total hip replacement patients were generally similar to those obtained in the previous studies. Although there was a flat dose-response relationship for the primary efficacy endpoint (p = 0.0852), there was a significant dose-response relationship for major venous thromboembolism (p = 0.0072). Nevertheless, a single oral dose of 30 mg was less effective than a single oral dose of 20 mg and increased the frequency of postoperative major bleeding events. In all groups receiving BAY 59-7939, major postoperative bleeding occurred upon the first postoperative dose and was significant (p = 0.0391) and dose related.

Taken together, the results of dose-finding studies of BAY 59-7939 lead to an interesting observation: it appears that higher doses increase the frequency of major postoperative bleeding events and are less effective in preventing venous thromboembolism compared to moderate doses. This could be by chance or because the anti-thrombotic effect is related to the strongest in situ inhibition of thrombin generated in the damaged vessel wall, which prevents local activation of protein C/thrombomodulin. The protein C anticoagulant pathway is a major system for controlling thrombosis and inflammation [30].

Thrombin binds to thrombomodulin and then activates protein C approximately 1000 times faster than free thrombin. Activated protein C proteolytically inactivates clotting factor Va and factor VIIIa, thereby blocking the amplification of the coagulation system [31].

Although thrombin increases protein C activation by binding to endothelial thrombomodulin, in atheroma, inflammation might overwhelm protective anticoagulant forces [32]. Inadequate amounts of residual thrombin due to therapy might prevent protein C from local activation, preventing the inactivation of factor Va and factor VIIIa by protein C. This in turn would allow activated factors V and factor VIII further activation of the clotting pathways with a local thrombus growing in one or more coronary, cerebrovascular, or peripheral artery atheroma. Thus, we can speculate that strong inhibition of local thrombin could paradoxically enhancing local thrombus formation instead of prevention.

The hypothesis that strong inhibition of thrombin could causes adverse outcomes is consistent with the physiological importance of activated protein C in the protein C anticoagulant pathway and in the control of the innate inflammatory response and because clinical studies reveal that deficiencies of protein C lead to microvascular thrombosis [30,33].

During treatment with antithrombotic drugs, in the presence of a wounded vessel, the relatively small amount of thrombin generated in situ is also inhibited by therapy. This can affect hemostasis with the potential consequence of bleeding and, although major bleeding could be a reversible event, antithrombotic therapy must be stopped which in turn could increase the risk of arterial thrombosis and death [34].

Platelets also play a central role in initiating and propagating pathological thrombosis.

Antiplatelet therapy is important in the management of acute coronary syndromes after spontaneous plaque disruption or mechanical disruption caused by percutaneous coronary intervention. Aspirin is the gold standard for prevention of acute myocardial syndromes, but higher dosages of aspirin do not provide better protection from events and are associated with increased risks of gastrointestinal bleeding [35].

The CURE trial [36] investigated the benefit of combined antiplatelet treatment (aspirin-clopidogrel) over aspirin alone in preventing thrombotic outcomes in patients with acute coronary syndrome. Major bleeding was significantly more common in the clopidogrel plus aspirin group (3.7%) versus the aspirin group (2.7%, p = 0.001). The sub-study of the CURE trial [37] showed that bleeding risks increase with increasing aspirin dose, without any increase in efficacy, and further increase with addition of clopidogrel to different doses of aspirin (Table 1). The CREDO trial evaluated the benefit of long-term treatment with clopidogrel in addition to aspirin therapy in patients after percutaneous coronary intervention. At 1 year there was an absolute reduction of 3% in the combined risk of death, MI, or stroke in favor of clopidogrel plus aspirin versus placebo plus aspirin. There was also a clear but nonsignificant difference in the risk of major bleeding (8.8% with clopidogrel-aspirin vs. 6.7% with placebo-aspirin; p = .07; absolute increase of 2.1%) [38]. The Charisma [3] study investigated, in patients with clinically evident cardiovascular disease or multiple risk factors, the benefit to receive clopidogrel (75 mg per day) plus low-dose aspirin (75 to 162 mg per day) or placebo plus low-dose aspirin. The use of clopidogrel-aspirin did not further reduce the incidence of the primary end point (myocardial infarction, stroke, or death from cardiovascular causes) compared to aspirin alone. However, considering the GUSTO definition, clopidogrel was associated with a significant increase in the rate of moderate bleeding or moderate and severe bleeding (Table 2). Although severe bleeding alone was not significantly greater with clopidogrel, a trend of concern was noted.

**Table 1:** Major bleeding with aspirin versus bleeding with clopidogrel plus aspirin.

Aspirin daily dose	Major bleeding		NNH Single/dual therapy
		
	Aspirin	Aspirin+ clopidogrel	
≤100 mg (%)	1.86	2.97	54/34
101–199 mg (%)	2.82	3.41	35/29
≥200 mg (%)	3.67	4.86	27/21
p =	<0.0001	< 0.001	

Major bleeding with aspirin versus bleeding with clopidogrel plus aspirin. Shown by NNH, bleeding risks increase with increasing aspirin dose and further increase with addition of clopidogrel to different doses of aspirin (Data from sub-study of the CURE trial [37]).

NNH: Number Needed to Harm

**Table 2:** Comparison of ischemic risk prevention and bleeding with clopidogrel plus aspirin (clop + ASA) versus aspirin plus placebo (ASA + placebo).

	Ischemic Events				Bleeding Events			
Trial (observation period)	clop + ASA (%)	ASA + placebo (%)	p	NNT	clop + ASA (%)	ASA + placebo (%)	p	NNH

Cure [36] (1 year)	9.3	11.4	<0.001	47.6	major 3.7 minor 5.1 total 8.5	major 2.7 minor 2.4 total 5.0	<0.001 <0.001 <0.001	100 37 28.6
Credo [38] (1 years)	8.5	11.2	0.02	37.0	severe 8.8 minor 5.3	severe 6.7 minor 5.6	0.07 0.92	250
Charisma [3] (2.5 years)	6.8	7.3	0.22	200	severe 1.7 moderate 2.1 moderate + severe 3.8	severe1.3 moderate 1.3 moderate + severe 2.6	0.09 <0.001 <0.001	250 125 83.3

Comparison of ischemic risk prevention and bleeding with clopidogrel plus aspirin (clop + ASA) versus aspirin plus placebo (ASA + placebo). Aspirin plus clopidogrel increased the incidence of bleeding events compared with aspirin plus placebo. Comparing the primary endpoint reduction with the two treatments, it appears that increased frequency of bleeding can limit the benefits of combined therapy.

NNT: Number Needed to Treat

NNH: Number Needed to Harm

The JUMBO-TIMI 26 trial [39] investigated a new antiplatelet drug, the thienopyridine P2Y_12 _ADP receptor antagonist prasugrel, in patients undergoing elective or urgent percutaneous coronary intervention. Patients were treated with a low dose (40 mg loading dose followed by 7.5 mg daily), an intermediate dose (60 mg loading dose followed by 10 mg daily), or a high dose (60 mg loading dose followed by 15 mg daily) of prasugrel or clopidogrel. Among prasugrel-treated patients, there were no group differences in the frequency of major adverse coronary events, although at 30 days myocardial infarction was less frequent in the high-dose group. At 30 days there was a trend toward a higher incidence of the composite of major, minor, or minimal bleeding for the high-dose prasugrel group (5.1%) compared to the intermediate and low-dose groups (3.5% with each). As expected, the vascular access site was the most frequent site of bleeding [39]. The JUMBO trial findings suggest that prasugrel is more potent than clopidogrel, but two episodes of profound platelet inhibition with prasugrel raise the possibility of a higher bleeding risk [40].

The reviewed studies suggest that when comparing low and high doses of antithrombotic drugs there is a strong dose-relationship regarding the capacity to prevent arterial thrombosis, but there is only a weak dose-relationship when comparing intermediate and high doses. However, there was a clear dose-related effect on bleeding risk in all trials. Thus, the highest doses of antithrombotic drugs yield the most frequent bleeding without providing the greatest level of prevention.

### A possible alternative to current antithrombotic therapy

**Figure 1:**
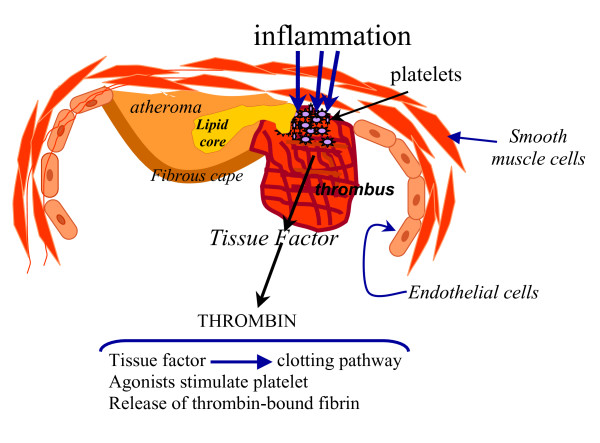
Thrombin generation in a disrupted atheroma. After plaque rupture, local inflammation leads to exposure of TF to the surrounding blood, initiating thrombin generation. Activated platelets release active components from the cytosol, which induces the externalization of phosphatidylserine through the flip-flop mechanism. Platelets exerts a regulatory function by serving as a source of inflammatory mediators and interacting with circulating white cells. Local blood flow changes in the culprit artery increase in situ prothrombotic conditions. Local fibrinolysis release thrombin-bound fibrin and contribute to thrombus growth.

At present, there is agreement that vessel wall inflammation is a major factor in the development of atherosclerosis, atheroma instability, and plaque disruption, events that are followed by local thrombosis, which underlies the clinical presentation of acute coronary syndromes. Inflammation is a reflexive response to mechanical irritation, or injury, and although restricted inflammation is beneficial, persistent inflammation incites tissue destruction [41]. In healthy subjects, the mean level of TF antigen in plasma is in the range of 149–172 pg/ml, whereas the levels of blood-borne TF are increased in atherosclerosis [42]. Plaque in unstable angina possesses elevated levels of TF that could be released during inflammation, increasing local thrombin generation and precipitating acute clinical syndromes. TF expressed by foam cells and in the necrotic core of plaque are higher in atheroma from patients with unstable angina compared with patients with stable angina [43]. Therefore, inflammation releasing TF, plays a pivotal role in the initiation of thrombotic complications in patients with coronary artery disease [44] (Figure 1).

In agreement with this, there is evidence that blocking inflammation can prevent thrombosis and acute coronary events. For example Versaci et al. [45] reported that corticosteroids have the potential to reduce the inflammatory response associated with stent implantation in patients with high C-reactive protein levels. The 12-month event-free survival rate was 93% and 65% in patients treated with prednisone and placebo, respectively. The 6-month restenosis rate was significantly lower for patients treated with prednisone versus those treated with placebo (7% vs. 33%, p = 0.001). Thus, prednisone reduces clinical events and the rate of angiographic restenosis.

Glucocorticoids can have rapid effects on inflammation but prolonged or high-dose glucocorticoid therapy has multiple side effects [46].

Altman et al.[47] (Table 3) reported findings from a pilot study showing that treatment with meloxicam, a preferential COX-2 inhibitor, is associated with a significant reduction in recurrent angina, non-fatal infarction, and cardiac death in patients with acute coronary syndromes without ST-segment elevation both in the care unit and after 3 months of follow-up. No adverse complications associated with meloxicam treatment were detected during the observation period.

**Table 3:** Results of the NUT study.

Events (%) at 3-month Follow-up	RR (%)	AR (%)	NNT
Patients (n)	60	60	
Recurrent angina + MI	58.6	28.3	3.5
Revascularization (PTCA and CABG)	61.0	18.3	5.5
Recurrent angina + MI + mortality	55.2	26.3	3.8
MI + revascularization + mortality	60.1	20	5.0

Results of the NUT study [34].

RR: Relative Reduction, AR: Absolute Reduction

NNT: Number Needed to Treat, MI: Myocardial Infarction.

There is accumulating evidence that the lipid-independent effects of cholesterol-lowering drugs are also relevant to the management of acute coronary syndromes. Statins exert a number of pleiotropic effects, including improvement of endothelial function, stabilization of atherosclerotic plaques, and inhibition of inflammatory responses. The anti-inflammatory effects of statins may have a clinical impact on vascular and nonvascular conditions such as multiple sclerosis and rheumatoid arthritis [48-50]. Pretreatment with atorvastatin 40 mg/day for 7 days significantly reduced procedural myocardial injury in patients undergoing elective coronary intervention [51]. In the ARMYDA-ACS trial, pretreatment of patients with non-ST-segment-elevation acute coronary syndrome with atorvastatin 80 mg 12 hr before percutaneous coronary intervention resulted in an 88% reduction in the relative risk of MACE at 30 days [52].

Atherothrombotic complications develop as a result of atherosclerosis in one or more coronary, cerebrovascular, or peripheral arteries. Is the thrombin generated in a disrupted atheroma similar in all patients? In the same patient is the thrombin generated in an inflamed plaque and a culprit lesion similar? Why are diabetic patients more resistant to the effects of antiplatelet drugs than are nondiabetic patients? [53].

**Figure 2:**
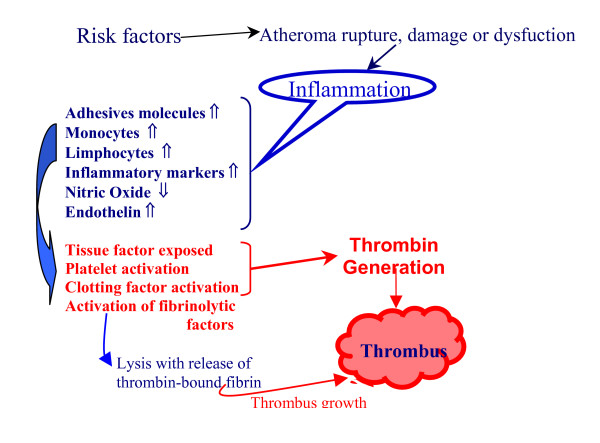
Reducing inflammation with anti-inflammatory drugs and lower doses of standard therapies (heparin, aspirin, clopidogrel, and/or new antithrombotic drugs, prasugrel, dabigatran, rivaroxaban) could diminish thrombin formation and prevent thrombus growth with less frequent bleeding. This represents a possible alternative to current antithrombotic therapy offering similar efficacy and less bleeding complications.

Based on the differences in the mechanisms underlying hemostasis and thrombosis, a practical point can be made regarding antithrombotic therapies. Although increased concentrations of antithrombotic drugs will affect both thrombosis and hemostasis, because thrombin generated in hemostasis is lower than in thrombosis, any increase in anticoagulant potential will produce a tendency toward bleeding [54]. Therefore, searching for alternative therapies in arterial thrombotic events suppressing inflammation with anti-inflammatory drugs could reduce thrombin formation in the disrupted atheroma lowering the need, of antithrombins or antiplatelet drugs (Figure 2).

However, in 2001, Mukherjee et al. [55] recommended caution in prescribing selective COX-2 inhibitors due to evidence for an increased risk of cardiovascular. The probable explanation for this finding is that selective inhibition of COX-2 reduces endothelial production of prostacyclin without affecting platelet production of TXA_2_, producing an imbalance in hemostatic prostanoids that might increase the risk for thrombotic cardiovascular events [56,57]. Reducing platelet production of TXA_2 _by the concomitant use of aspirin might offset the prothrombotic effect of selective COX-2 inhibitor. and other NSAID-related MI risk.

As mentioned above, preliminary data from our randomized controlled trial of the preferential COX-2 inhibitor meloxicam in patients with acute coronary syndrome who were treated with aspirin demonstrated a statistically significant reduction in cardiovascular events [42] and no clinical complications during the study period. Also, analyses have established that C-reactive protein, a marker of inflammatory activation, is a significant predictor of adverse cardiovascular events in patients with atherosclerosis, independent of baseline characteristics and treatments [58]. It was showed that coadministration of the selective COX-2 inhibitor rofecoxib with low-dose aspirin decreases inflammatory and oxidative indices, attenuating C-reactive protein and interleukin-6 [59,60].

Very recently Koo et al. present the report on the use of the COX-2 inhibitor celecoxib, to reduce restenosis of patients underwent coronary angioplasty. After 6 months paclitaxel-eluting stent implantation mean in-stent late luminal loss was lower in the celecoxib group (0.49 mm, SD 0.47) than in the control group (0.75 mm, SD 0.60, absolute difference 0.26 mm; 95% CI 0.12–0.40). Also celecoxib reduced the need for revascularisation of the target lesion [61].

In conclusion, the beneficial preventive effect of high doses of a single antithrombotic drug or combined antithrombotic therapy could be overcome by the tendency toward bleeding. and there is a dose-related association between bleeding and death [62]. Determining the appropriate balance between preventing ischemic events and causing bleeding in these patients presents a challenging problem for clinicians [62], because in everyday clinical practice, patients may be at greater risk of major bleeding and ischemic events.

Thus, the preferred approach might be to use anti-inflammatory drugs in addition to antithrombin drugs and aspirin to reduce inflammation and thrombin formation in atheroma. Although some preliminary data have been already published, in acute coronary syndromes large prospective double-bind randomized trials are necessary to confirm the potential benefit of this treatment.

## References

[B1] Altman R, Scazziota AS, Herrera ML, Gonzalez C (2006). Thrombin generation by activated factor VII on platelet activated by different agonists. Extending the cell-based model of hemostasis Thromb J.

[B2] Aledort LM (2000). Recombinant factor VII: Ia is a pan-hemostatic agent?. Thromb Haemost.

[B3] Bhatt DL, Fox KA, Hacke W, Berger PB, Black HR, Boden WE, Cacoub P, Cohen EA, Creager MA, Easton JD, Flather MD, Haffner SM, Hamm CW, Hankey GJ, Johnston SC, Mak KH, Mas JL, Montalescot G, Pearson TA, Steg PG, Steinhubl SR, Weber MA, Brennan DM, Fabry-Ribaudo L, Booth J, Topol EJ, CHARISMA Investigators (2006). Clopidogrel and aspirin versus aspirin alone for the prevention of atherothrombotic events. N Engl J Med.

[B4] Ballew KA (2006). Clopidogrel plus aspirin did not differ from aspirin alone for reducing MI, stroke, and CV death in high risk atherothrombosis. Evid Based Med.

[B5] Fisher M, Lees K, Norris JW, Barnett HJ (2006). CHARISMA: The Antiplatelet Saga Continues. Stroke.

[B6] Mohler ER (2007). Atherothrombosis – Wave Goodbye to Combined Anticoagulation and Antiplatelet Therapy?. N Engl J Med.

[B7] Doggrell SA (2003). Warfarin and aspirin give more benefit than aspirin alone but also more bleeding after myocardial infarction. Expert Opin Pharmacother.

[B8] Fox KA, Mehta SR, Peters R, Zhao F, Lakkis N, Gersh BJ, Yusuf S, for Clopidogrel in Unstable Angina to Prevent Recurrent Ischemic Events Trial (2004). Benefits and risks of the combination of clopidogrel and aspirin in patients undergoing surgical revascularization for non-ST-elevation acute coronary syndrome: the Clopidogrel in Unstable Angina to Prevent Recurrent Ischemic Events (CURE) Trial. Circulation.

[B9] Eikelboom JW, Hirsh J (2006). Bleeding and Management of Bleeding. Eur Heart J.

[B10] Colman RW (2006). Are hemostasis and thrombosis two sides of the same coin?. J Exp Med.

[B11] Renne T, Pozgajova M, Gruner S, Schuh K, Pauer HU, Burfeind P, Gailani D, Nieswandt B (2005). Defective thrombus formation in mice lacking coagulation factor XII. J Exp Med.

[B12] Moreno PR, Purushothaman KR, Fuster V, Echeverri D, Truszczynska H, Sharma SK, Badimon JJ, O'Connor WN (2004). Plaque neovascularization is increased in ruptured atherosclerotic lesions of human aorta: implications for plaque vulnerability. Circulation.

[B13] Furie B, Furie BC (2007). In vivo thrombus formation. J Thromb Haemost.

[B14] Day SM, Reeve JL, Pedersen B, Farris DM, Myers DD, Im M, Wakefield TW, Mackman N, Fay WP (2005). Macrovascular thrombosis is driven by tissue factor derived primarily from the blood vessel wall. Blood.

[B15] Hoffman M, Whinna HC, Monroe DM (2006). Circulating tissue factor accumulates in thrombi, but not in hemostatic plugs. J Thromb Haemost.

[B16] Bach RR (2006). Tissue factor encryption. Arterioscl Thromb Vasc Biol.

[B17] Hemker HC, Béguin S (1995). Thrombin generation in plasma: its assessment via the endogenous thrombin potential. Thromb Haemost.

[B18] Niemetz J, Fallon JT, Harrington E, Hathcock J (2004). Rapid generation of thrombin by atheroma and platelets. J Thromb Haemost.

[B19] Schroeder V, Chatterjee T, Mehta H, Windecker S, Pham T, Devantay N, Meier B, Kohler HP (2002). Thrombin activatable fibrinolysis inhibitor (TAFI) levels in patients with coronary artery disease investigated by angiography. Thromb Haemost.

[B20] Gurfinkel E, Altman R, Scazziota A, Rouvier J, Mautner B (1994). Importance of thrombosis and thrombolysis in silent ischaemia: comparison of patients with acute myocardial infarction and unstable angina. Br Heart J.

[B21] The Thrombolysis in Myocardial Infarction (TIMI) 11A Trial Investigators (1997). Dose-Ranging Trial of Enoxaparin for Unstable Angina: Results of TIMI 11A. J Am Coll Cardiol.

[B22] FRISC Study Group (1996). Low molecular weight heparin (Fragmin^®^) during instability in coronary artery disease (FRISC). Lancet.

[B23] Eriksson BI, Arfwidsson AC, Frison L, Eriksson UG, Bylock A, Kalebo P, Fager G, Gustafsson D (2002). A dose-ranging study of the oral direct thrombin inhibitor, ximelagatran, and its subcutaneous form, melagatran, compared with dalteparin in the prophylaxis of thromboembolism after hip or knee replacement: METHRO I. MElagatran for THRombin inhibition in Orthopaedic surgery. Thromb Haemost.

[B24] Albers GW, Diener HC, Frison L (2005). Ximelagatran vs warfarin for stroke prevention in patients with nonvalvular atrial fibrillation: a randomized trial. JAMA.

[B25] Petersen P, Grind M, Adler J, SPORTIF II Investigators Ximelagatran versus warfarin for stroke prevention in patients with nonvalvular atrial fibrillation (2003). SPORTIF II: a dose-guiding, tolerability, and safety study. J Am Coll Cardiol.

[B26] Eriksson BI, Dahl OE, Büller HR, Hettiarachchi R, Rosencher N, Bravo M-L, Ahnfelt L, Piovella F, Stangier J, Kälebo P, Reilly P (2005). A new oral direct thrombin inhibitor, dabigatran etexilate, compared with enoxaparin for prevention of thromboembolic events following total hip or knee replacement: the BISTRO II randomized trial. J Thromb Haemost.

[B27] Turpie AGG, Fisher WD, Bauer KA, Kwong LM, Irwin MW, Kälebo P, Misselwitz F, Gent M, For the Odixa-Knee study group (2005). BAY 59-7939: an oral, direct Factor Xa inhibitor for the prevention of venous thromboembolism in patients after total knee replacement. A phase II dose-ranging study. J Thromb Haemost.

[B28] Eriksson BI, Borris L, Dahl OE, Haas S, Huisman MV, Kakkar AK, Misselwitz F, Kälebo P (2006). Oral, direct Factor Xa inhibition with BAY 59-7939 for the prevention of venous thromboembolism after total hip replacement. J Thromb Haemost.

[B29] Eriksson BI, Borris LC, Dahl OE, Haas S, Huisman MV, Kakkar AK, Muehlhofer E, Dierig C, Misselwitz F, Kalebo P, ODIXa-HIP Study Investigators (2006). A once-daily, oral, direct Factor Xa inhibitor, rivaroxaban (BAY 59-7939), for thromboprophylaxis after total hip replacement. Circulation.

[B30] Esmon CT (2003). The protein C pathway. Chest.

[B31] Esmon CT (2006). Inflammation and the activated protein C anticoagulant pathway. Semin Thromb Hemost.

[B32] Spronk HM, van der Voort D, Ten Cate H (2004). Blood coagulation and the risk of atherothrombosis: a complex relationship. Thromb J.

[B33] Esmon CT (2004). Crosstalk between inflammation and thrombosis. Maturitas.

[B34] Segev A, Strauss BH, Tan M, Constance C, Langer A, Goodman SG, Canadian Acute Coronary Syndromes Registries Investigators (2005). Predictors and 1-year outcome of major bleeding in patients with non-ST-elevation acute coronary syndromes: insights from the Canadian Acute Coronary Syndrome Registries. Am Heart J.

[B35] Campbell CL, Smyth S, Montalescot G, Steinhubl SR (2007). Aspirin Dose for the Prevention of Cardiovascular Disease: A Systematic Review. JAMA.

[B36] Yusuf S, Zhao F, Mehta SR, Chrolavicius S, Tognoni G, Fox KK, The Clopidogrel in Unstable Angina to Prevent Recurrent Events Trial Investigators (2001). Effects of clopidogrel in addition to aspirin in patients with acute coronary syndromes without ST-segment elevation. N Engl J Med.

[B37] Peters RJ, Mehta SR, Fox KA, Zhao F, Lewis BS, Kopecky SL, Diaz R, Commerford PJ, Valentin V, Yusuf S, Clopidogrel in Unstable angina to prevent Recurrent Events (CURE) Trial Investigators (2003). Effects of Aspirin Dose When Used Alone or in Combination With Clopidogrel in Patients With Acute Coronary Syndromes: Observations From the Clopidogrel in Unstable angina to prevent Recurrent Events (CURE) Study. Circulation.

[B38] Steinhubl SR, Berger PB, Mann JT, Fry ET, DeLago A, Wilmer C, Topol EJ, CREDO Investigators (2002). Clopidogrel for the Reduction of Events During Observation. Early and sustained dual oral antiplatelet therapy following percutaneous coronary intervention: a randomized controlled trial. JAMA.

[B39] Wiviott SD, Antman EM, Winters KJ, Weerakkody G, Murphy SA, Behounek BD, Carney RJ, Lazzam C, McKay RG, McCabe CH, Braunwald E, JUMBO-TIMI 26 Investigators (2005). Randomized comparison of prasugrel (CS-747, LY640315), a novel thienopyridine P2Y12 antagonist, with clopidogrel in percutaneous coronary intervention: results of the Joint Utilization of Medications to Block Platelets Optimally (JUMBO)-TIMI 26 trial. Circulation.

[B40] Serebruany VL, Midei MG, Meilman H, Malinin AI, Lowry DR (2006). Platelet inhibition with prasugrel (CS-747) compared with clopidogrel in patients undergoing coronary stenting: the subset from the JUMBO study. Postgrad Med J.

[B41] Rhen T, Cidlowski JA (2005). Antiinflammatory action of glucocorticoids – new mechanisms for old drugs. N Engl J Med.

[B42] Mackman N (2004). Role of Tissue Factor in Hemostasis, Thrombosis, and Vascular Development. Arterioscler Thromb Vasc Biol.

[B43] Annex BH, Denning SM, Channon KM, Sketch MH, Stack RS, Morrissey JH, Peters KG (1995). Differential expression of tissue factor protein in directional atherectomy specimens from patients with stable and unstable coronary syndromes. Circulation.

[B44] Vojacek J, Dusek J, Bis J, Stasek J, Blazek M Plasma tissue factor in coronary artery disease. Further step to the understanding of the basic mechanisms of coronary artery thrombosis. Physiol Res.

[B45] Versaci F, Gaspardone A, Tomai F, Ribichini F, Russo P, Proietti I, Ghini AS, Ferrero V, Chiariello L, Gioffre PA, Romeo F, Crea F, Immunosuppressive Therapy for the Prevention of Restenosis after Coronary Artery Stent Implantation Study (2002). Immunosuppressive therapy for the prevention of restenosis after coronary artery stent implantation (IMPRESS Study). J Am Coll Cardiol.

[B46] Schacke H, Docke WD, Asadullah K (2002). Mechanisms involved in the side effects of glucocorticoids. Pharmacol Ther.

[B47] Altman R, Luciardi HL, Muntaner J, Del Rio F, Berman SG, Lopez R, Gonzalez C (2002). Efficacy Assessment of Meloxicam, a Preferential Cyclooxygenase-2 Inhibitor, in Acute Coronary Syndromes Without ST-Segment Elevation. The Nonsteroidal Anti-Inflammatory Drugs in Unstable Angina Treatment-2 (NUT-2) Pilot Study. Circulation.

[B48] Chello M, Anselmi A, Spadaccio C, Patti G, Goffredo C, Di Sciascio G, Covino E (2007). Simvastatin increases neutrophil apoptosis and reduces inflammatory reaction after coronary surgery. Ann Thorac Surg.

[B49] Ray KK, Cannon CP (2007). Lipid-independent Pleiotropic Effects of Statins in the Management of Acute Coronary Syndromes. Curr Treat Options Cardiovasc Med.

[B50] Wu YS, Hu YY, Yang RF, Wang Z, Wei YY (2007). The matrix metalloproteinases as pharmacological target in osteoarthritis: Statins may be of therapeutic benefit. Med Hypotheses.

[B51] Pasceri V, Patti G, Nusca A, Pristipino C, Richichi G, Di Sciascio G, ARMYDA Investigators (2004). Randomized trial of atorvastatin for reduction of myocardial damage during coronary intervention: results from the ARMYDA (Atorvastatin for Reduction of MYocardial Damage during Angioplasty) study. Circulation.

[B52] Patti G, Pasceri V, Colonna G, Miglionico M, Fischetti D, Sardella G, Montinaro A, Di Sciascio G (2007). Atorvastatin pretreatment improves outcomes in patients with acute coronary syndromes undergoing early percutaneous coronary intervention. J Am Coll Cardiol.

[B53] Angiolillo DJ, Shoemaker SB, Desai B, Yuan H, Charlton RK, Bernardo E, Zenni MM, Guzman LA, Bass TA, Costa MA (2007). Randomized Comparison of a High Clopidogrel Maintenance Dose in Patients With Diabetes Mellitus and Coronary Artery Disease Results of the Optimizing Antiplatelet Therapy in Diabetes Mellitus (OPTIMUS) Study. Circulation.

[B54] Alexander KP, Chen AY, Roe MT, Newby LK, Gibson CM, Allen-LaPointe NM, Pollack C, Gibler WB, Ohman EM, Peterson ED, CRUSADE Investigators (2005). Excess dosing of antiplatelet and antithrombin agents in the treatment of non-ST-segment elevation acute coronary syndromes. JAMA.

[B55] Mukherjee D, Nissen SE, Topol EJ (2001). Risk of cardiovascular events associated with selective COX-2 inhibitors. JAMA.

[B56] Fitzgerald GA (2004). Coxibs and cardiovascular disease. N Engl J Med.

[B57] Antman EM, Bennett JS, Daugherty A, Furberg C, Roberts H, Taubert KA, American Heart Association (2007). Use of nonsteroidal antiinflammatory drugs: an update for clinicians: a scientific statement from the American Heart Association. Circulation.

[B58] Sabatine MS, Morrow DA, Jablonski KA, Rice MM, Warnica JW, Domanski MJ, Hsia J, Gersh BJ, Rifai N, Ridker PM, Pfeffer MA, Braunwald E, PEACE Investigators (2007). Prognostic significance of the Centers for Disease Control/American Heart Association high-sensitivity C-reactive protein cut points for cardiovascular and other outcomes in patients with stable coronary artery disease. Circulation.

[B59] Bogaty P, Brophy JM, Noel M, Boyer L, Simard S, Bertrand F, Dagenais GR (2004). Impact of prolonged cyclooxygenase-2 inhibition on inflammatory markers and endothelial function in patients with ischemic heart disease and raised C-reactive protein: a randomized placebo-controlled study. Circulation.

[B60] Monakier D, Mates M, Klutstein MW, Balkin JA, Rudensky B, Meerkin D, Tzivoni D (2004). Rofecoxib, a COX-2 inhibitor, lowers C-reactive protein and interleukin-6 levels in patients with acute coronary syndromes. Chest.

[B61] Koo BK, Kim YS, Park KW, Yang HM, Kwon DA, Chung JW, Hahn JY, Lee HY, Park JS, Kang HJ, Cho YS, Youn TJ, Chung WY, Chae IH, Choi DJ, Oh BH, Park YB, Kim HS (2007). Effect of celecoxib on restenosis after coronary angioplasty with a Taxus stent (COREA-TAXUS trial): an open-label randomised controlled study. Lancet.

[B62] Eikelboom JW, Mehta SR, Anand SS, Xie C, Fox KA, Yusuf S (2006). Adverse impact of bleeding on prognosis in patients with acute coronary syndromes. Circulation.

